# Energy harvesting efficiency of piezoelectric polymer film with graphene and metal electrodes

**DOI:** 10.1038/s41598-017-17791-3

**Published:** 2017-12-11

**Authors:** Sanghoon Park, Yura Kim, Hyosub Jung, Jun-Young Park, Naesung Lee, Yongho Seo

**Affiliations:** 1 Graphene Research Institute, Sejong University, Seoul, 05006 Republic of Korea; 2 Faculty of Nanotechnology & Advanced Materials Engineering and HMC, Sejong University, Seoul, 05006 Republic of Korea

## Abstract

In this study, we investigated an energy harvesting effect of tensile stress using piezoelectric polymers and flexible electrodes. A chemical-vapor-deposition grown graphene film was transferred onto both sides of the PVDF and P(VDF-TrFE) films simultaneously by means of a conventional wet chemical method. Output voltage induced by sound waves was measured and analyzed when a mechanical tension was applied to the device. Another energy harvester was made with a metallic electrode, where Al and Ag were deposited by using an electron-beam evaporator. When acoustic vibrations (105 dB) were applied to the graphene/PVDF/graphene device, an induced voltage of 7.6 V_pp_ was measured with a tensile stress of 1.75 MPa, and this was increased up to 9.1 V_pp_ with a stress of 2.18 MPa for the metal/P(VDF-TrFE)/metal device. The 9 metal/PVDF/metal layers were stacked as an energy harvester, and tension was applied by using springs. Also, we fabricated a full-wave rectifying circuit to store the electrical energy in a 100 μF capacitor, and external vibration generated the electrical charges. As a result, the stored voltage at the capacitor, obtained from the harvester via a bridge diode rectifier, was saturated to ~7.04 V after 180 s charging time.

## Introduction

Energy harvesting technology, which converts natural dissipating energy to usable energy, has been studied to help mitigate energy depletion around the globe, and thus various methods to harvest this energy have been suggested^1–4^. In particular, energy harvesting technology using piezoelectric materials is one such method which utilizes mechanical energy from various sources such as human motion, acoustic noise, or wind to convert energy into an electric current or voltage^5^. When mechanical energy such as an acoustic wave is applied to the piezoelectric polymer film, electrical charges are induced between the two surfaces. Using this property, a piezoelectric material can be applied as an electromechanical energy converter^6^. Some polymers have strong piezoelectric effects when subjected to mechanical stretching or external excitation. Thus, piezoelectric polymers can be adopted as a generator material for energy harvesting devices as well as actuators, and received great attention from researchers for this reason. As one of the representative piezoelectric materials, polyvinylidene fluoride **(**PVDF) films with high sensitivity and flexibility have been studied for application in an energy harvesting device^7^.

Some conducting materials with flexibility have been adopted as electrodes for PVDF devices, and among them graphene in particular has been considered widely^8,9^. Graphene has been studied as a transparent electrode material due to its outstanding physical properties including high electrical conductivity, high thermal conductivity, and optical transparency^9–13^. Graphene has been considered as an alternative material to replace indium tin oxide thin film as a transparent electrode. In recent times, many research groups have used graphene as electrodes for energy harvesting devices because of its flexibility and stretchability. In this study, we fabricated a PVDF film based piezoelectric generator with graphene electrodes on both sides of the PVDF. This PVDF generator using thin metal film electrodes was made with a metal/PVDF/metal (M/PVDF/M) structure to enable comparisons of the output power of the device with graphene/PVDF/graphene (G/PVDF/G). A higher output power was obtained from M/PVDF/M devices than that from G/PVDF/G devices.

Besides the electrode issues generators, previous studies on PVDF based generators have been conducted by only applying external sources such as acoustic noise, vibration, wind, etc., to the generator. Furthermore, while using acoustic noise as an external source, we applied a continuous tensile stress to the aforementioned generators by hanging weights. As a result, we were able to obtain a remarkably high output voltage from generators by gradually increasing the continuous tension. Also, by mounting the generator on a stretchable frame with compressed springs, we were able to boost the output voltage by means of the tensile stress. External sources consisting of both acoustic noise and mechanical shocks were used to vibrate the sample. In addition to these experiments, we also prepared copolymer polyvinylidene fluoride-trifluoroethylene [P(VDF-TrFE)] based generators, and measured their performance based on the thickness of the piezoelectric film in the same manner in the aforementioned experimental process. It was observed that a thin P(VDF-TrFE) generator induced a higher power density than that generated by a thick film.

## Experiment

### Piezoelectric polymer film fabrication

**Figure 1:**
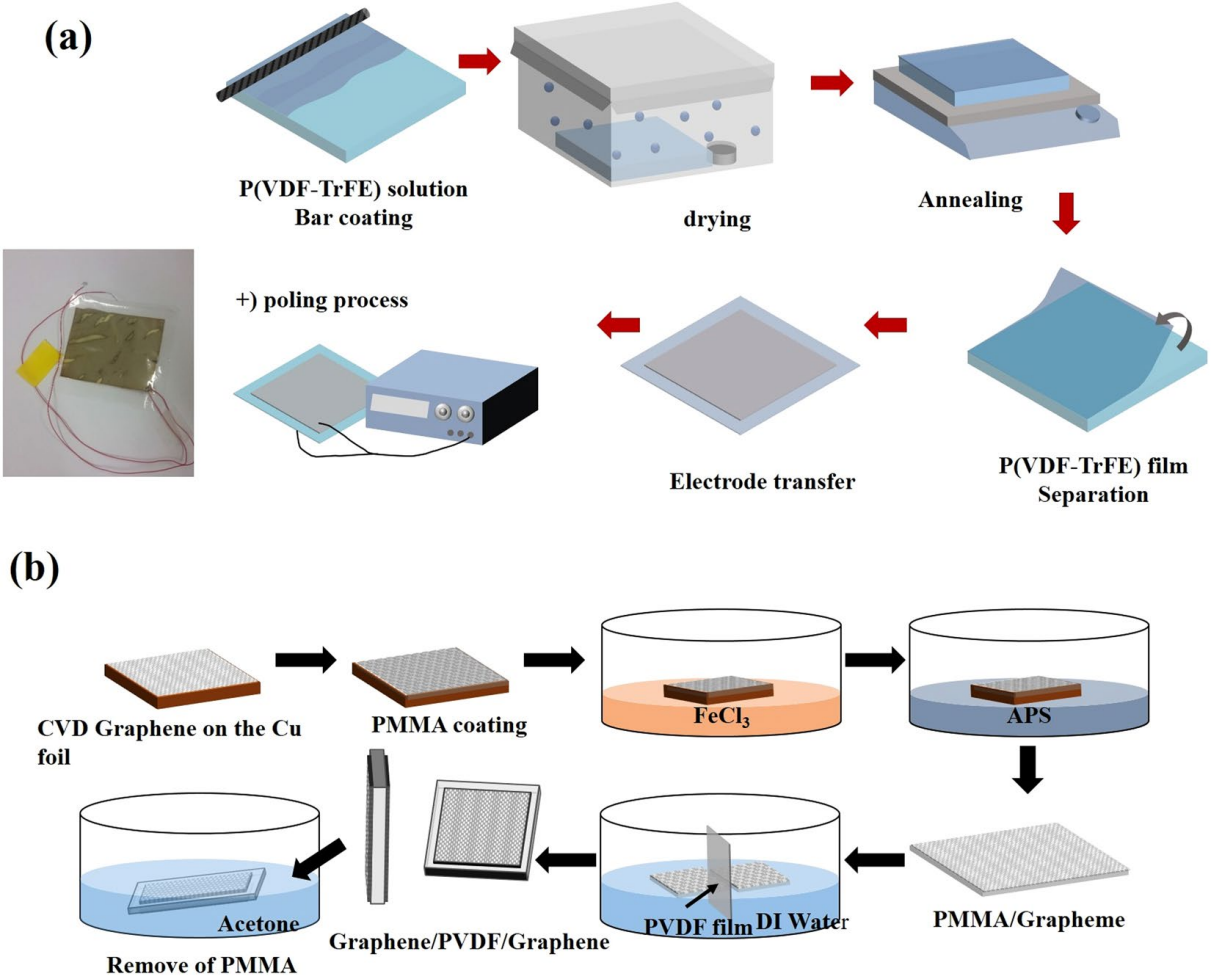
Schematics of the fabrication process for (**a**) P(VDF-TrFE) based thin film generators (the inset shows a photograph of a fabricated device with silver electrodes), and (**b**) a G/PVDF/G generator fabricated by double-sided graphene transfer.

To fabricate the piezoelectric film, P(VDF-TrFE) powder (Piezotech Co.) was dissolved into N,N-dimethylformamide (DMF) with 15 wt%, and a bar coating method was employed to coat the solution onto a silicon wafer with 300 nm oxide. After that, the sample was slowly dried in a closed chamber filled with DMF solvent for two hours to reduce the surface roughness of the P(VDF-TrFE) film and make it denser to improve performance^14^. To evaporate the remaining solvent, the sample was annealed at 60 °C for 40 minutes, and additional annealing (at 140 °C for 2 hours) followed successively to enhance the piezoelectric property created through the phase change of P(VDF-TrFE) from α to β-phase^15^. After the annealing steps, the sample was dipped into DI water to detach the hydrophobic P(VDF-TrFE) film from the substrate, and a P(VDF-TrFE) film with ~10 μm thickness was obtained. Then, as a poling process to align the polarizations, an electric field of 50 MV/m was applied to the film. All processes are depicted in Fig. 1(a). A readily available commercial film (Fils Co., Ltd., 80 μm thickness) was used for the thick PVDF film device.

### Electrode deposition

The graphene sheets were synthesized by a conventional chemical vapor deposition process^16^. The copper substrate holding graphene sheets was wet etched before the transfer step by the same process already explained in our previous work^17^. When we originally tried to transfer graphene layers on to both sides of the PVDF film, we attempted to transfer them one at a time, but the first layer became easily detached during the second layer transfer due to weak adhesion between the graphene and the polymer film. To solve this problem, two graphene sheets were transferred simultaneously as shown in Fig. 1(b). First they were floated on DI water, then a PVDF film or a P(VDF-TrFE) film was dipped into the water and placed vertically between both of the graphene sheets. As the piezoelectric film was raised slowly in a vertical position, the graphene sheets were attached to both sides of the film simultaneously. The samples were hanged immediately after transfer to flow down the water and annealed at 100 °C to remove residual water. The PMMA layer was removed by acetone after drying the sample completely. An aluminum (or silver) electrode was deposited on both sides of the PVDF and P(VDF-TrFE) films via an electron-beam evaporator. It was found that either Al or Ag indicated similar performance as electrodes for the piezoelectric device, because they have similar work functions^18–20^. The thickness of the metal film was controlled to 50 nm which would maintain the flexibility of the energy harvester. The active areas were about 6 × 6.5 cm^2^ for the PVDF and 4 × 4 cm^2^ for the P(VDF-TrFE) samples, respectively.

### Experimental Setup

To isolate external background noise and building vibrations, a soundproof chamber and a pneumatic vibration isolation table were used. The fast Fourier transform from detected signals was employed to confirm the frequency spectrum of the background noise of the setup. Though no intentional excitations were applied, a peak was observed at 60 Hz (See Supplementary Fig. S1). This peak is attributed to the frequency of the utility power, and no other significant frequency was observed, confirming that this setup was isolated from external vibrations and background noise.

**Figure 2:**
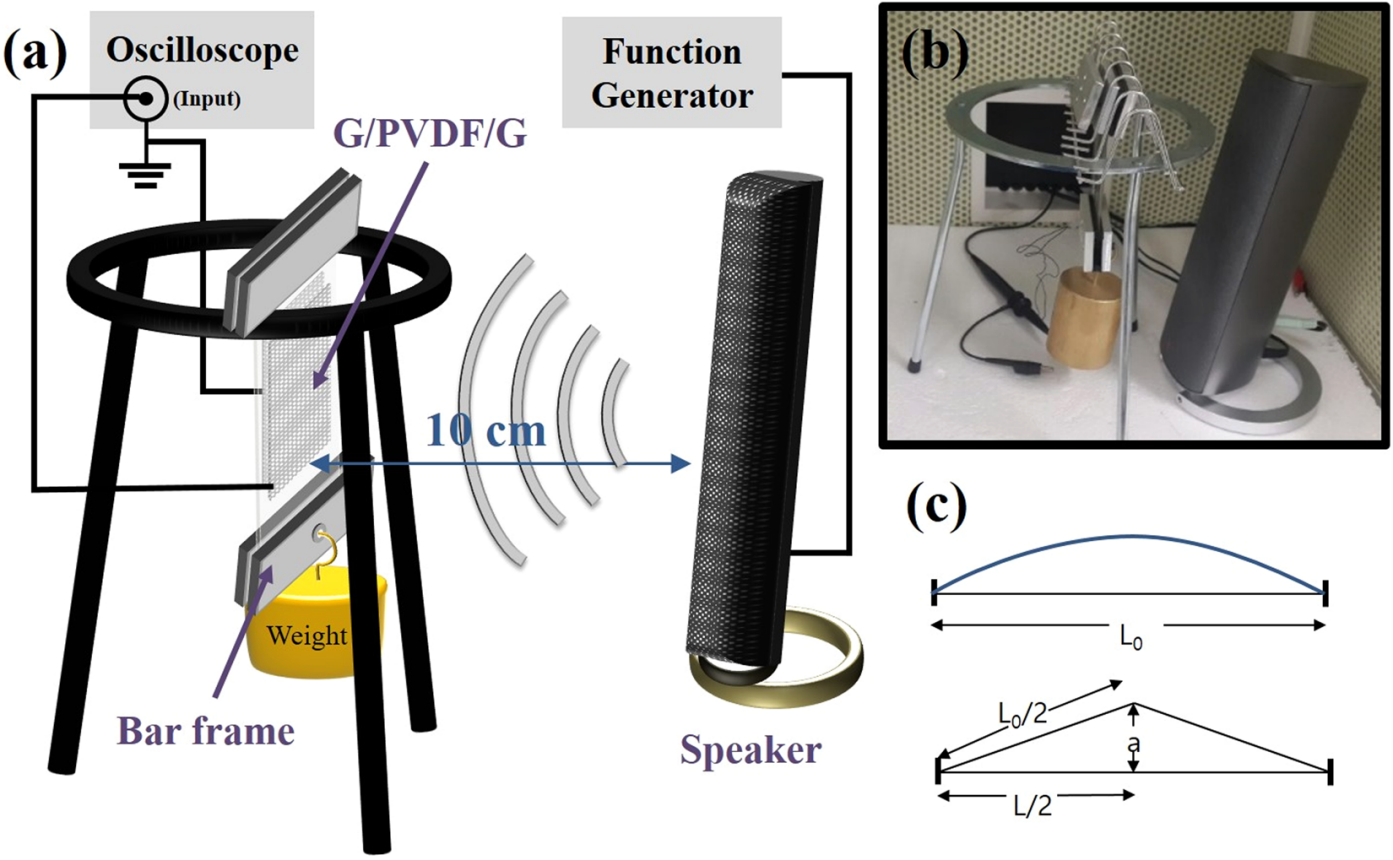
(**a**) Schematics of experimental setup applying tensile stress to the generator. The output voltage was measured by an oscilloscope. (**b**) Photograph shows the stressed film based generator and a loudspeaker. (**c**) Illustration shows the model of the vibration of the film with fixed ends.

The top and bottom edges of the rectangular film was fixed with two bar frames and was suspended from a tripod, as shown in Fig. 2(a). The bar frame at the bottom weighed about 70 g and additional weights were hung on to apply longitudinal tension due to gravitational force. The tensile stress was controlled by changing weights. The acoustic vibration was excited by means of a loudspeaker. Its frequency and amplitude were controlled by a function generator. The frequency was in the range of 100 Hz to 300 Hz, and the excitation amplitude was fixed at 5 V. Loudness at 10 cm distance was measured as 105 to 110 dB by a decibel meter, with variations depending on the frequency. (See Supplementary Fig. S2) The induced voltage was measured by an oscilloscope with an input impedance 1 MΩ.

## Result and Discussion

### Effect of tensile stress on piezo-film

The required mechanical energy to vibrate the stressed film can be approximately estimated by the work done by pulling the film a distance of *a* as shown in Fig. 2(c). Here, the film is stressed by tension $F=\sigma {A}_{c}$, where σ is tensile stresses, and ${A}_{c}=t{w}_{d}$ is the cross sectional area of the film (*t*: thickness, *w*_*d*_: width). Considering the film as an elastic medium, the spring constant *k* of the film is given by $k=E{A}_{c}/{L}_{0}$, where *E* is Young’s modulus. The length change of the film, ${\rm{\Delta }}L=L-{L}_{0}$, can be approximated as ${\rm{\Delta }}L\cong \frac{2{a}^{2}}{{L}_{0}}$, assuming $L\cong {L}_{0}$. The work done by pulling, *W* is

1 $$W=F\,{\rm{\Delta }}L\cong \sigma {A}_{c}\frac{2{a}^{2}}{{L}_{0}}.$$

When we apply a mechanical pulse to excite the films with different thicknesses *t*, the vibration amplitude (*a*) is proportional to $1/\sqrt{t}$ from Eq. (1) for the same supplied energy cost. The vibrating piezo-film induces charge *Q*, which can be estimated from;

2 $$Q={d}_{31}EA\varepsilon ,$$

where *d*_31_ is the piezoelectric constant, and *A* = *Lw*_*d*_ and $\varepsilon =\frac{{\rm{\Delta }}L}{{L}_{0}}$ is the strain. The electrical energy converted from the mechanical work can be described by the electric energy stored in the capacitor ${W}_{s}=\frac{1}{2}{Q}^{2}/C$, where the capacitance C = $\frac{{\varepsilon }_{d}\,A}{t}$, and ${\varepsilon }_{d}$ is the permittivity of the piezo-film. Therefore, the stored energy is given by

3 $${W}_{s}=\frac{t{{d}_{31}}^{2}{E}^{2}{\varepsilon }^{2}A}{2{\varepsilon }_{d}}.$$

Finally, the energy conversion ratio *η* is given by,

4 $$\eta =\frac{{W}_{s}}{W}\cong \frac{{{L}_{0}}^{2}{{d}_{31}}^{2}{E}^{2}{\varepsilon }^{2}}{4{\varepsilon }_{d}{\rm{\sigma }}{a}^{2}}.$$

As a preliminary experiment to confirm the flexibility of graphene electrodes in piezoelectric generation, we compared the induced voltages of devices with silver paste and graphene as electrodes on PVDF film. A signal almost ten times greater was measured from graphene coated devices as graphene was flexible and did not damp the vibrational motion of the PVDF film (See Supplementary Fig. S3).

**Figure 3:**
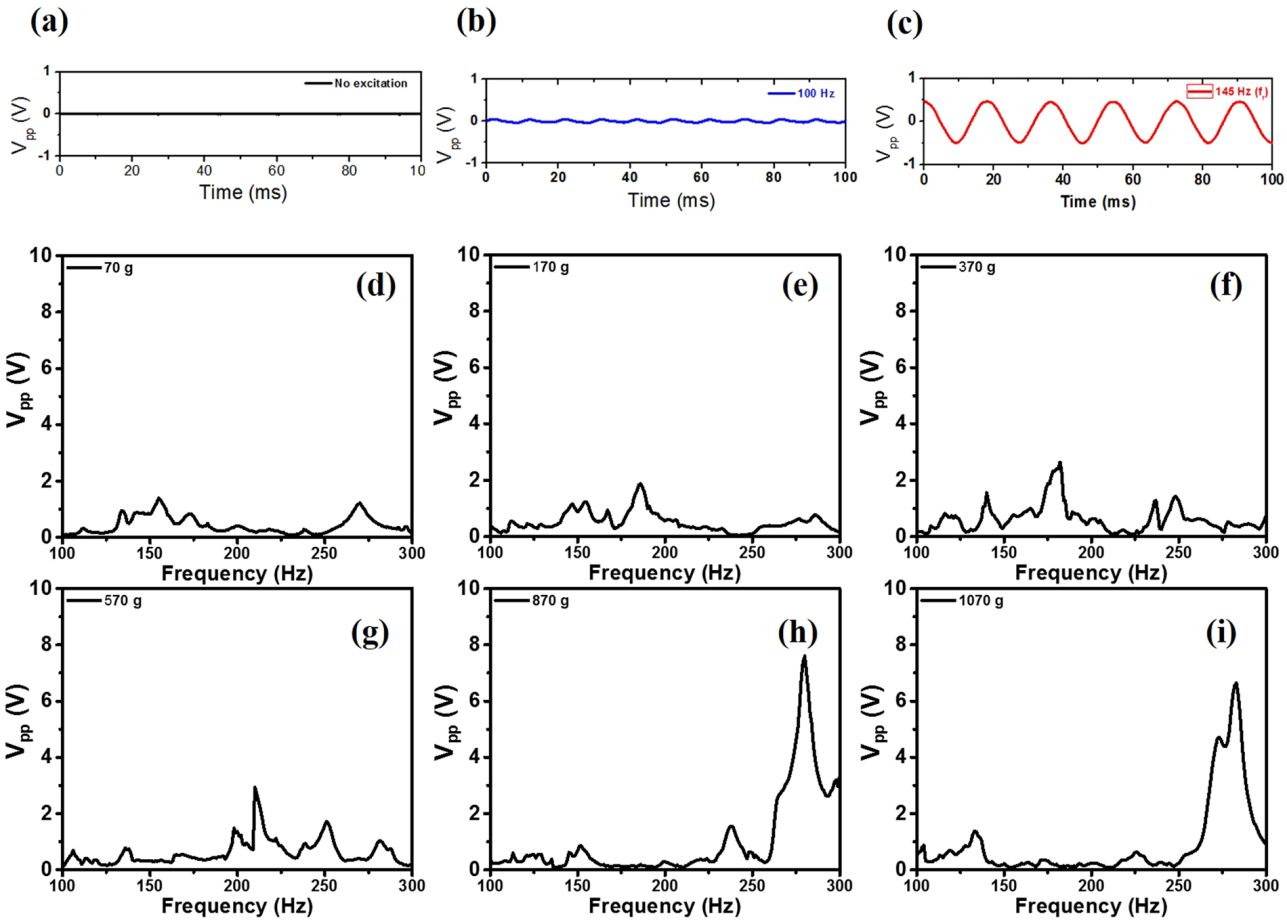
The waveforms of the detected voltages from the stretched G/PVDF/G generator under 1.2 MPa stress were investigated, depending on different acoustic environments. (**a**) No acoustic excitation was applied intentionally, and a small noise signal (20 mV_pp_) with 60 Hz was detected. (**b**) When the acoustic excitation (86.7 dB) was applied at 100 Hz, the device generated 95 mV_pp_. (**c**) When the acoustic wave (83.7 dB) at resonance frequency (220 Hz) was applied, 1.12 V_pp_ was detected. After that, the input voltage was increased to 5 V, and the peak-to-peak output voltage spectra were measured under tensile stresses by different weights: (**d**) 70 g, (**e**) 170 g, (**f**) 370 g, (**g**) 570 g, (**h**) 870 g, and (**i**) 1070 g.

The waveforms of the detected piezoelectric voltages under different acoustic environments were investigated as shown in Fig. 3(a–c). (a) When no acoustic excitation was applied intentionally a very small signal (20 mV_pp_) at 60 Hz was detected. In that situation, an acoustic sound of 48 dB was measured in the soundproof chamber. (b) When the acoustic wave (86.7 dB) was excited by an input value of 1.0 V and 100 Hz from the function generator, the detected signal increased to 95 mV_pp_. (c) When the acoustic wave (83.7 dB) was excited by the same voltage but at the resonance frequency ƒ_r_ (220 Hz), an extremely high voltage 1.12 V_pp_ was generated. The result shows that the generator was active at under weak acoustic excitation conditions with an off-resonance frequency and even without an intentional source if it is stretched by the static stress.

The peak-to-peak voltages were measured as a function of frequency with higher excitation (~105 dB) by 1 V input as shown in Fig. 3(d–i). (d) When a low tension (70 g) was applied to the device to make it flat, its maximum peak-to-peak voltage (V_pp_) was found to be 1.4 V at 155 Hz. In this graph, there was no significant resonant behavior. The data at low tension exhibited multiple peaks by spurious modes due to its crumpled surface, but it was suppressed by the reinforced tension. When we applied higher tensile stress to the device by hanging heavier weights, the piezo-film was pulled tight. The maximum voltages at its resonance frequencies ƒ_r_ were measured as (e) 1.9 V_pp_ at 185 Hz, (f) 2.6 V_pp_ at 182 Hz, (g) 2.9 V_pp_ at 210 Hz, (h) 7.6 V_pp_ at 280 Hz, and (i) 6.6 V_pp_ at 283 Hz, respectively, and the applied masses were 170 g, 370 g, 570 g, 870 g and 1070 g, respectively. It is notable that the measured voltage depends on the load resistance. When an 870 g weight was applied, a maximum voltage of 7.6 V_pp_ was detected. This voltage is more than 20 times higher than that measured by others^21^ at similar conditions^22^, and it is comparable to the voltages obtained by a bending or stretching motion of the piezo-film^14,21,23^. Considering the input impedance 1 MΩ of the oscilloscope in this measurement, the voltage 7.6 V_pp_ corresponds to a generating power 7.2 μW. To confirm the reproducibility of the spectra data, we repeated the measurements with the same samples and obtained very consistent results (See Supplementary Fig. S4).

### Effect of electrodes on piezo-film

**Figure 4:**
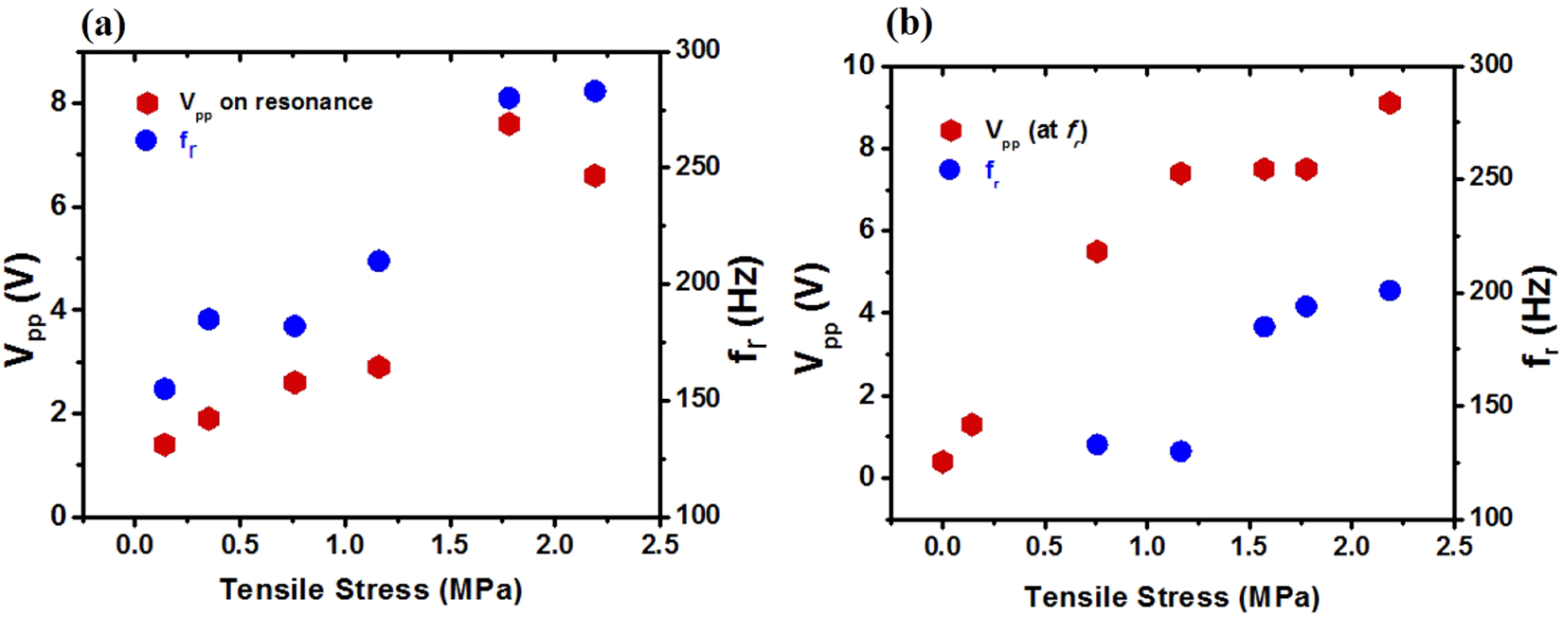
The resonance frequency ƒ_r_ and the voltage V_pp_ at ƒ_r_ were plotted as functions of the stress for two different samples: (**a**) G/PVDF/G device and (**b**) M/PVDF/M device. The left axis indicates the peak-to-peak voltage scale, and the right shows the resonance frequencies.

The fundamental vibrational mode of a stretched string with fixed ends is that the wavelength is twice the length of the string, and the resonance angular frequency for the standing wave is given by^24^

5 $${{\rm{\omega }}}_{r}=\frac{\pi }{{L}_{0}}\sqrt{\frac{\sigma }{\rho }},$$

where linear density $\rho =m/{L}_{0}$, *L*_0_ is the original length of the film. When there is no stress on the film, no resonance is expected, and the resonance frequency is supposed to be proportional to the square root of *σ* from Eq. (5). The measured resonance frequency ƒ_r_ and the voltage V_pp_ at ƒ_r_ were plotted as functions of the stress in Fig. 4, where the tensile stresses were calculated from ${\rm{\sigma }}={F}_{g}/{A}_{c}$ with the gravitational forces *F*_*g*_ created by weights. The V_pp_ and ƒ_r_ appeared to rise as the tensile stress *σ* was increased. This increasing tendency of the resonance frequency can be explained by the stretched string model from Eq. (5), but the measured frequencies were roughly twice the calculated values. This discrepancy is attributed to the difference of the geometric shape of the piezo-film as it is a film instead of a string, because Eq. (5) was derived from 1-dimensional string model. The influence of the electrodes was ignored as the electrode thickness (50 nm for metal film, 0.3 nm for graphene) was too thin to have a considerable spring constant, compared with the piezo-film (80 μm for commercial film, ~10 μm for synthesized film). The relationship between the amplitude and the applied tension can be explained by the increment of the quality factor, as well as the resonance frequency. From a damped harmonic oscillator model^24^, the quality factor *Q* is given by $Q={\omega }_{r}/2\beta$, where *β* is a damping parameter. The amplitude at the resonance frequency is proportional to the frequency and inversely proportional to damping. As the film was stretched by tensile stress, the internal friction was reduced and the film’s quality factor was increased. Additionally, the application of a large tensile strain could increase the β-phase portion in PVDF film^25^. The abrupt changes of the data points between 1.2 and 1.75 MPa in Fig. 4(a) are attributed to a mode mixing between the fundamental mode and other spurious modes. It is notable that the applied tension increases the vibration amplitude at high frequencies and it also augments the piezoelectric energy harvesting effect.

The same measurements were performed for the sample (M/PVDF/M) with metal electrodes instead of graphene electrodes, applying tensile stress as shown in Fig. 4(b). (For details see Supplementary Fig. S5). The stress dependencies of V_pp_ and f_r_ for both G/PVDF/G and M/PVDF/M devices were similar, but the output voltage for M/PVDF/M was slightly higher than that for G/PVDF/G. The metal electrode generator exhibited an output voltage 1.94 V higher on average than the graphene electrode generator at the same tensile stress. The high piezoelectric generation for metal electrodes can be explained by the fact that the transferred graphene has residues on the surface and trapped water, but evaporated metal film has a cleaner surface with good electrical contact. Particularly, in the case of the M/PVDF/M device, the maximum voltage 0.4 V_pp_ without tensile stress was increased up to 9.1 V_pp_ with the stress at 2.18 MPa, corresponding to the areal power density 0.27 μW/cm^2^, which was much higher than that of G/PVDF/G (0.18 μW/cm^2^). This phenomenon also can be explained by the resonance with the enhanced quality factor which is caused by the blue shift of the resonance frequency and diminished internal friction.

### Effect of thickness of piezo-film

**Figure 5:**
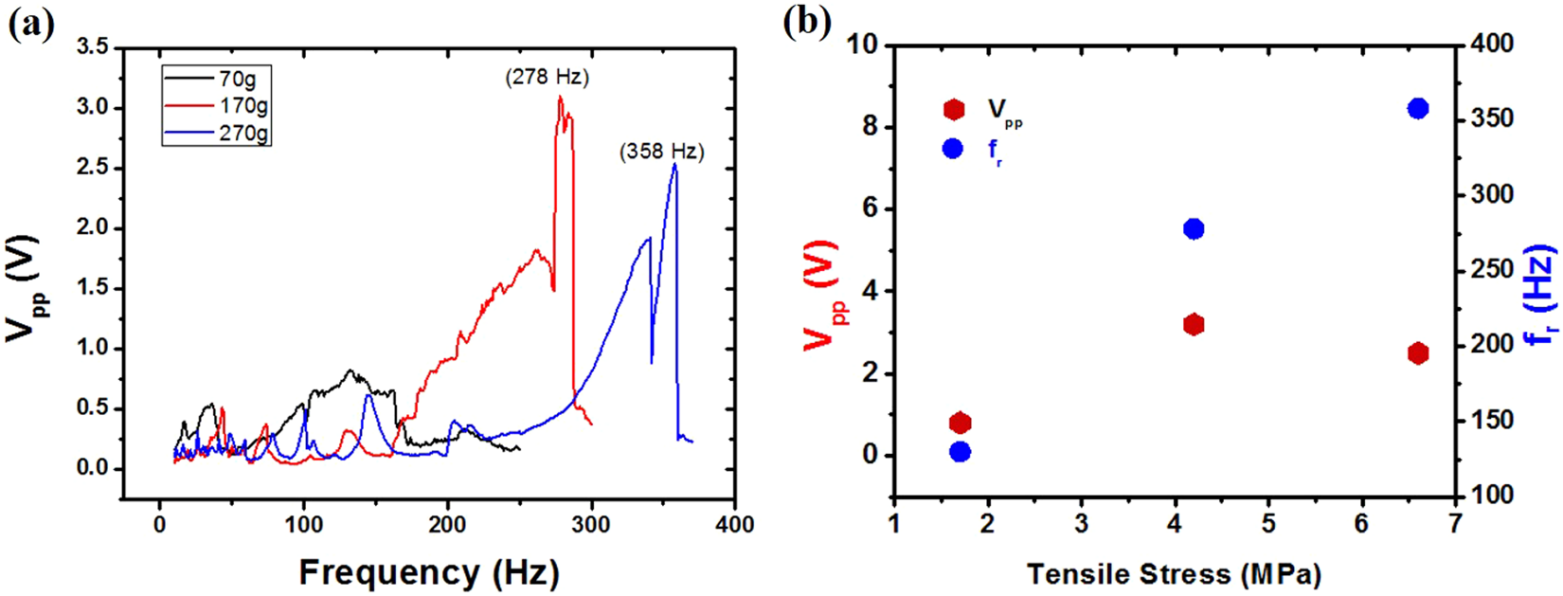
(**a**) The generated peak-to-peak voltages from the device based on P(VDF-TrFE) thin film were measured as a function of frequency. (**b**) The peak-to-peak voltage and resonance frequency were plotted as functions of the tensile stress.

The same measurements were carried out for the thin film device based on P(VDF-TrFE) film, and similar results were obtained as shown in Fig. 5. While no sharp peak was found by loading a light weight (70 g), the voltages have peaks at 278 Hz and 358 Hz for loading masses 170 g (4.2 MPa) and 270 g (6.6 MPa), respectively. For this thin film, high stresses were applied with low weights, and the stressed film has high resonance frequencies due to the light mass of the film. The induced voltages were lower than those of the thick film device, but the generated power density of the thin film device was higher. The advantage of the thin piezo-film was studied by Sung *et al*. previously, and the higher output voltage was obtained from a thinner film^25^. In our experiment, the maximum power density for the thin film device was estimated as 81.3 W/m^3^ at 4.2 MPa, while the maximum power for the thicker PVDF film was 33.8 W/m^3^ at 2.2 MPa. This result is a remarkably high value in comparison to other piezoelectric polymer devices^9^. On the other hand, the tensile strain ${\rm{\varepsilon }}=\sigma /E$ was estimated as 0.132% under the maximum stress $\sigma =6.6$ MPa, where Young’s modulus *E* of the piezo-film (~ 5 GPa). In this estimation, the influence of electrodes on the strain was neglected because of its ignorable thickness. The static strain in our experiment is comparable to the dynamic strain of the other group’s piezoelectric generator (0.083–0.271%) reported by Lee *et al*.^21^. It is notable that the static strain can be kept without supplying additional input energy, but the dynamic strain costs kinetic energy. From Eq. (4), the unknown parameter *ε* can be replaced by the term including input energy from Eq. (4), and the energy conversion ratio for a single piezo layer,

6 $$\eta \cong \frac{{{d}_{31}}^{2}{E}^{2}}{{\varepsilon }_{d}{{\rm{\sigma }}}^{2}{w}_{d}{L}_{0}}\frac{W}{t}$$

is inversely proportional to the thickness if one supplies the same amount of mechanical energy for the finite tolerable stress. In Eq. (6), the conversion ratio is only a function of thickness, which means that the ratio can be enhanced by making the film thin. Furthermore, the total energy efficiency can be boosted by stacking thin film layers.

### Creating an energy harvester by stacking piezo-films

**Figure 6:**
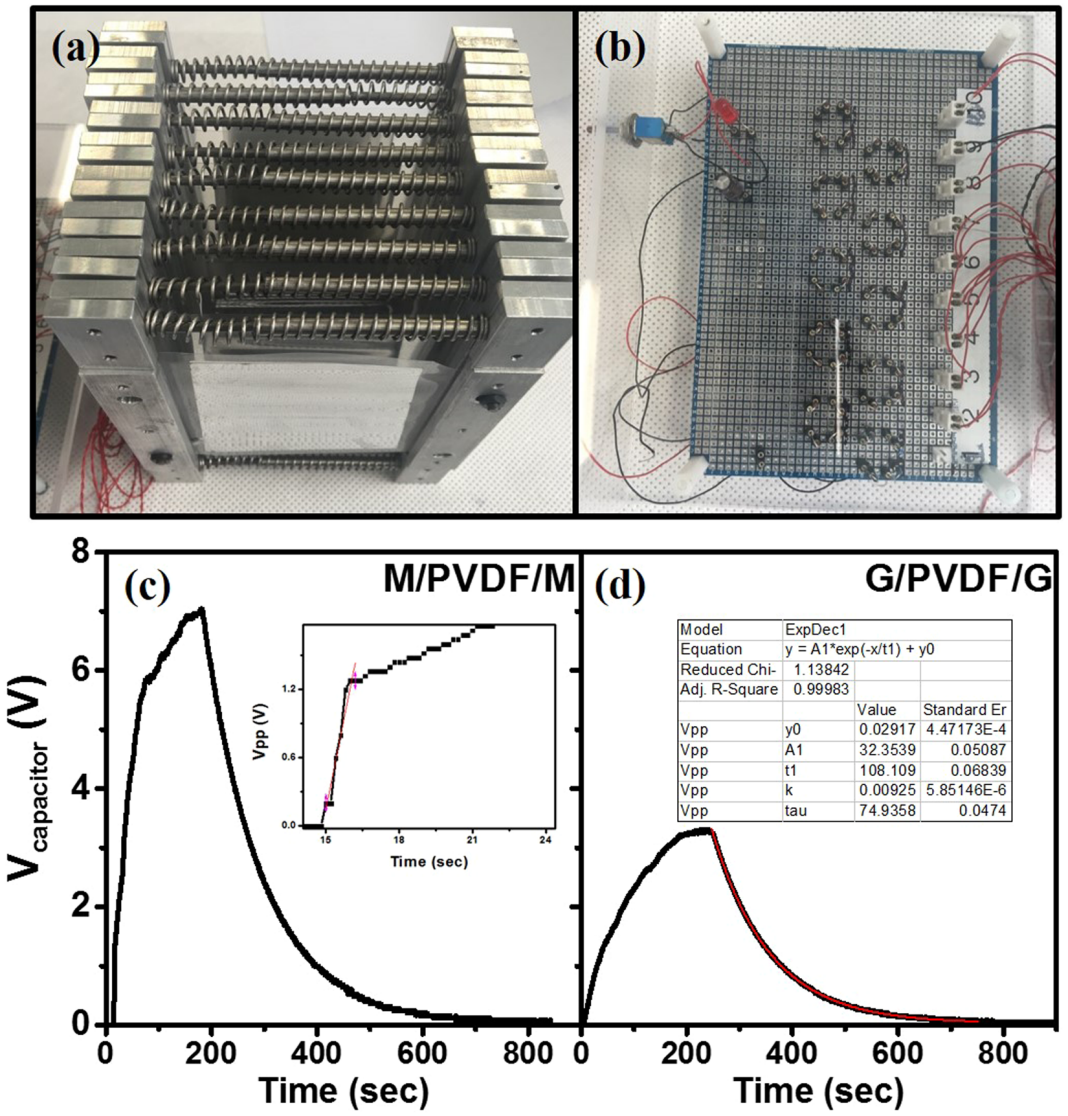
(**a**) Photograph of stacked generators in parallel connection. (**b**) The rectifier circuit to accumulate the output voltage. The voltage changes over time during the charging and discharging processes are shown for the generators with (**c**) metal electrodes and (**d**) graphene electrodes.

Tensile stress was finally applied by using square frames with compressed springs, as shown in Fig. 6a. We clamped the left and right sides of the piezo-film, and two compressed springs (1.03 kN/m spring constant) were inserted to apply tensile stress. Its 10 mm contraction caused F_t_ = 20.6 N tension, and the tensile stress σ and strain ε were 4.29 MPa and 0.086%, respectively. A bridge-diode rectifier circuit (Fig. 6b) for charging the capacitor using four diodes (BAV20) was built with the generators to demonstrate its performance. Mechanical tapping was exploited as an energy source.

Nine clamped generators (*N* = 9) were stacked and electrically connected in parallel and we applied mechanical pulses repeatedly. As we applied a single mechanical pulse in every second by tapping, expansion or contraction of the film ΔL ~ 0.015 mm occurred, and a few volts in open circuit were generated on the piezo-film. We continued to apply mechanical pulses until the capacitor (100 μF) was saturated, and then left it to observe the leakage current. In Fig. 6c and d, the capacitor was saturated to 7.04 V at 180 s from the metal electrodes generators, and 3.3 V at 240 s was shown in regard to graphene electrode generators. From the initial charging rate (inset in Fig. 6c), the work done by a single mechanical pulse (*W* = *N F*_*t*_ *ΔL*) was calculated to be 2.78 mJ and the stored energy $({W}_{s}=\frac{1}{2}C{V}^{2})$ in the capacitor was 57 μJ. Thus, approximately 2.05% of maximum conversion efficiency was obtained from the stressed piezo-film stacked generator. Even though this efficiency is similar to others^26^, it should be noted that this efficiency was obtained from a final system including a rectifying circuit. During the discharging process, the time constant *τ* was estimated as 108 s by fitting into an exponential decay function. Particularly, the leakage currents $({{\rm{I}}}_{leak}\cong \frac{Q}{\tau }\cong 6.5\,\mu {\rm{A}})$ through the diodes and electrolyte capacitor were dominant factors in reducing the conversion efficiency. In terms of electrodes, the metal electrodes were more effective than the graphene electrodes due to their surface cleanness and good electrical contact.

## Conclusion

This study showed that the performance and sensitivity of an energy harvesting device composed of representative piezoelectric polymer materials can be improved by applying proper tensile stress. Also, our experimental results indicate that thin piezoelectric film exhibits better energy conversion efficiency. The thin piezo-film was more effective in converting acoustic waves into electrical charge because free standing thin film is more flexible and more sensitive to the acoustic waves. The thin piezoelectric P(VDF-TrFE) film (~10 μm) with uniform roughness was fabricated successfully, and this showed good tolerance to tensile stress of up to 6.6 MPa. The maximum power density for the thin film device was estimated as 81.3 W/m^3^ at 4.2 MPa. A higher output power was obtained from devices with metallic electrodes than those with graphene electrodes. A theoretical model to calculate the conversion ratio of the energy harvesting devices was suggested.

In summary, we suggest a novel technique with tensile stress to enhance the performance of PVDF or P(VDF-TrFE) based energy harvesting devices with high output power. The energy conversion ratio can be enhanced by making the piezo-film thin. This novel solution can be employed not only for PVDF or P(VDF-TrFE) but also for other flexible piezoelectric materials in the field of energy harvesting technology. The uniform thin piezoelectric film with application of suitable tensile stress can be utilized for a commercialized energy harvesting device.

## Electronic supplementary material

Supplementary information
