# Supplementary material for: Energy harvesting efficiency of piezoelectric polymer film with graphene and metal electrodes
Source: Sci Rep. 2017 Dec 11;7:17290. doi: 10.1038/s41598-017-17791-3 (PMC5725581; doi:10.1038/s41598-017-17791-3)
Supplement: Supplementary file 1 — Supplementary information [file 41598_2017_17791_MOESM1_ESM.pdf]

Supplementary Information

Energy harvesting efficiency of piezoelectric polymer film with graphene and metal electrodes

Sanghoon Park[1§], Yura Kim[1§], Hyosub Jung[1§], Jun-Young Park[2], Naesung Lee[2], and Yongho Seo[1,2*]

[1]Graphene Research Institute, Sejong University, Seoul 05006, Republic of Korea

[2]Faculty of Nanotechnology & Advanced Materials Engineering and HMC,

Sejong University, Seoul 05006, Republic of Korea

[*] Corresponding author: yseo@sejong.ac.kr

[§]equally contributed

CONTENTS:

1. Background noise test
2. Loudness of the acoustic excitation.
3. Performance test of graphene electrodes
3. Experimental reproducibility test.
4. Metal electrode PVDF generator
5. SEM, XRD and Optical microscope images of P(VDF-TrFE)
7. Calculation of the length change by a mechanical pulling

1. Background noise test

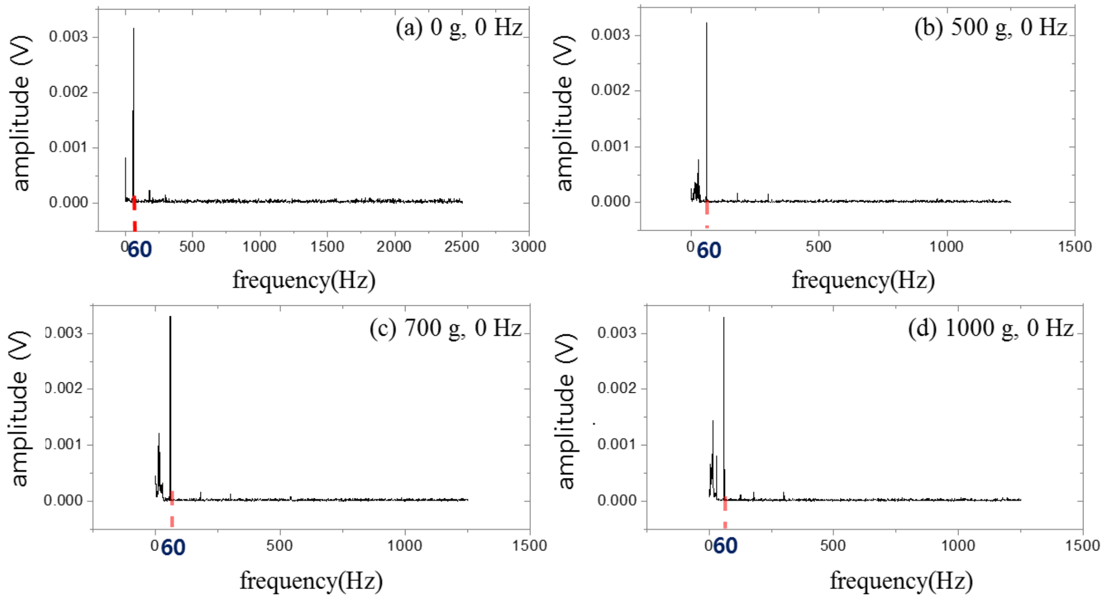

Fig. S1 The FFT analysis was employed to confirm the frequency spectrum of the background noise of the setup to distinguish internal and external vibrations. A main peak was observed at 60 Hz while no intentional excitation were applied.

FFT (fast Fourier transform) from the signal measured by an oscilloscope was analyzed to identify the characteristics of the signal. Though there was no external stimulus in the sample, some peaks were found under different tensile forces applied to the sample. The signal at 60 Hz is not related with a piezoelectric effect but an electrical noise caused by a utility power. Except this peak, minor peaks were observed at low frequency range, which are presumably irregular mechanical shocks from the building. No significant peaks found at frequencies higher than 100 Hz. As a result, it has been proved that the chamber are prevented from vibrating from the floor or outside by means of the vibration damping unit.

2. Loudness of the acoustic excitation.

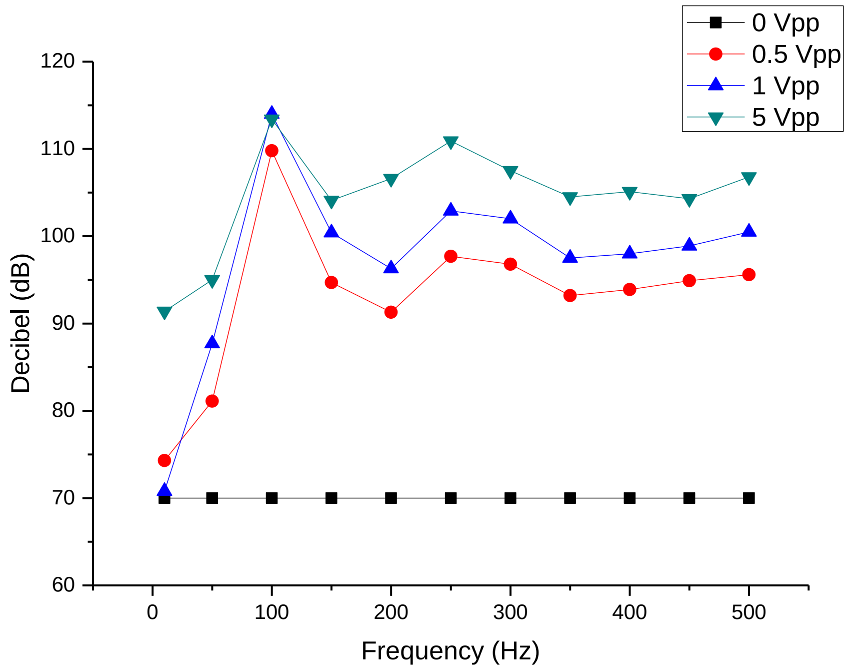

Fig. S2 The loudness of the acoustic excitation from a loudspeaker was measured at the distance 10 cm by a decibel meter.

3. Performance test of graphene electrodes

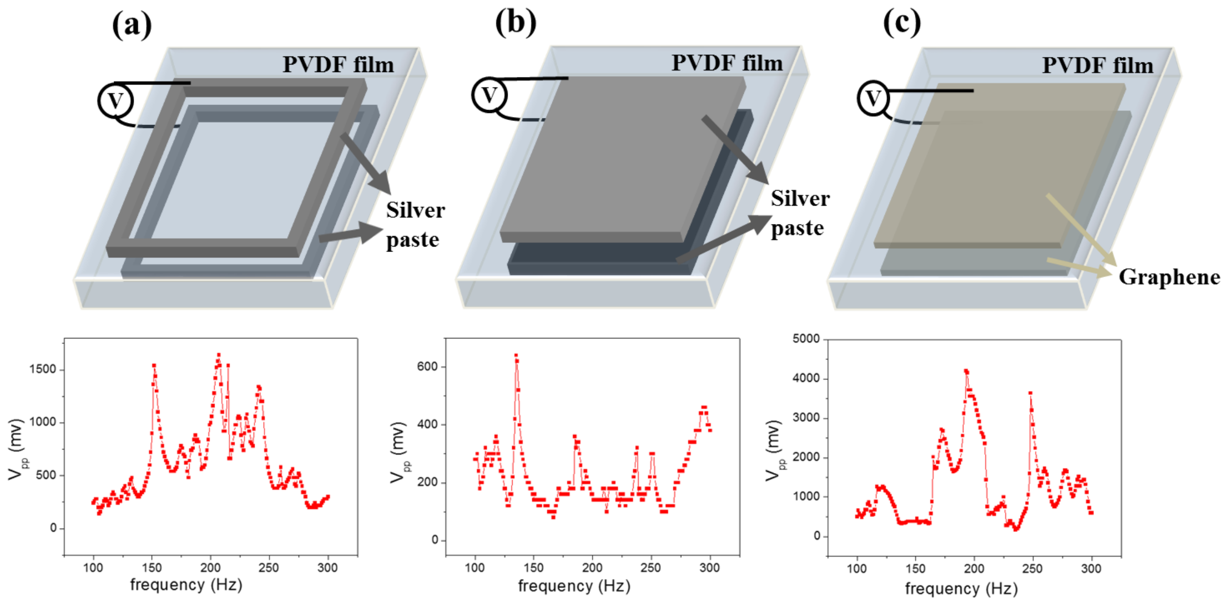

Fig. S3 To confirm the active area of piezoelectric generation, we compared a peak-to-peak voltage (V_{pp}) of devices which have silver paste electrodes (a) only at edge of PVDF film and (b) on full area. (c) Graphene was transferred as electrode, and the same measurement was performed.

The test for the performance of the graphene electrode was carried out by comparing three different samples. The first one was covered by silver paste only at the border of the PVDF film (Fig. S3a) and second one was covered with silver paste on all rectangular area (Fig. S3b) and the last one was coated with graphene sheets (Fig. S3c). The same tensile stress (induced by 500 g) and external acoustic source (frequency range of 100~300 Hz) were applied. The highest peak-to-peak voltages at f_r was 1.65 V_{pp}, 643 mV_{pp} and 4.17 V_{pp}. The graphene electrodes device showed almost 2.5 times as high as those for silver paste electrodes devices. The main reason for reduced signal in devices with silver pastes is that the thick silver paste hindered the PVDF film from vibrating freely. This confirmed the usefulness of graphene as electrodes for the transparent and flexible energy harvesting device.

4. Repeatability test

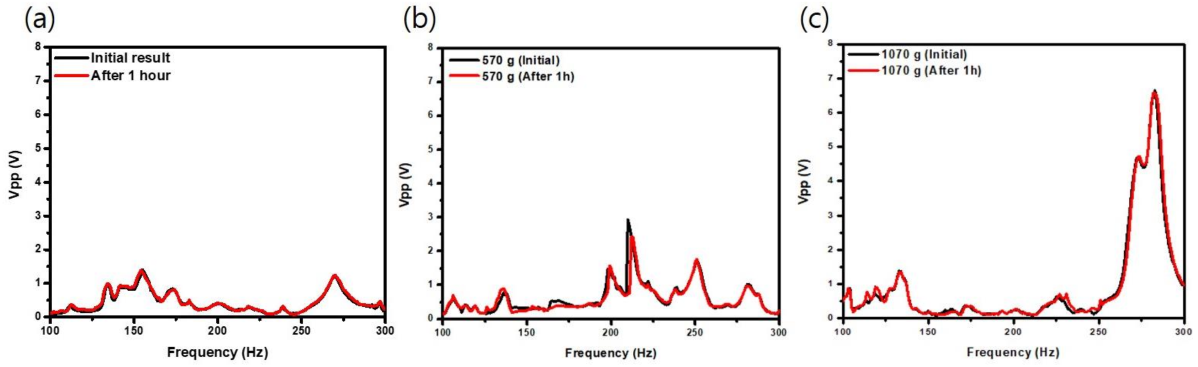

Fig. S4 Repeatability test shows highly reproducible measurement of the generator.

The output voltage according to the tension applied to the energy harvester were measured twice. The tension was controlled by the weights. Figure S4(a) shows the maximum voltage 1.5 V at 155 Hz when the tension was not applied. Figure S4(b) shows the maximum voltage 2.9 V at 210 Hz when the tension is applied with 570 g weight. Figure S4(c) shows the maximum voltage 6.7 V at 280 Hz with 1070 g. Even though the curves seems arbitrary, second measurement (red) after 1 hr showed almost the same curves. This complicated spectral form presumably originated from the spurious modes due to the non-uniformity of the film shape and its crumpled surface.

5. Metal electrode PVDF generator

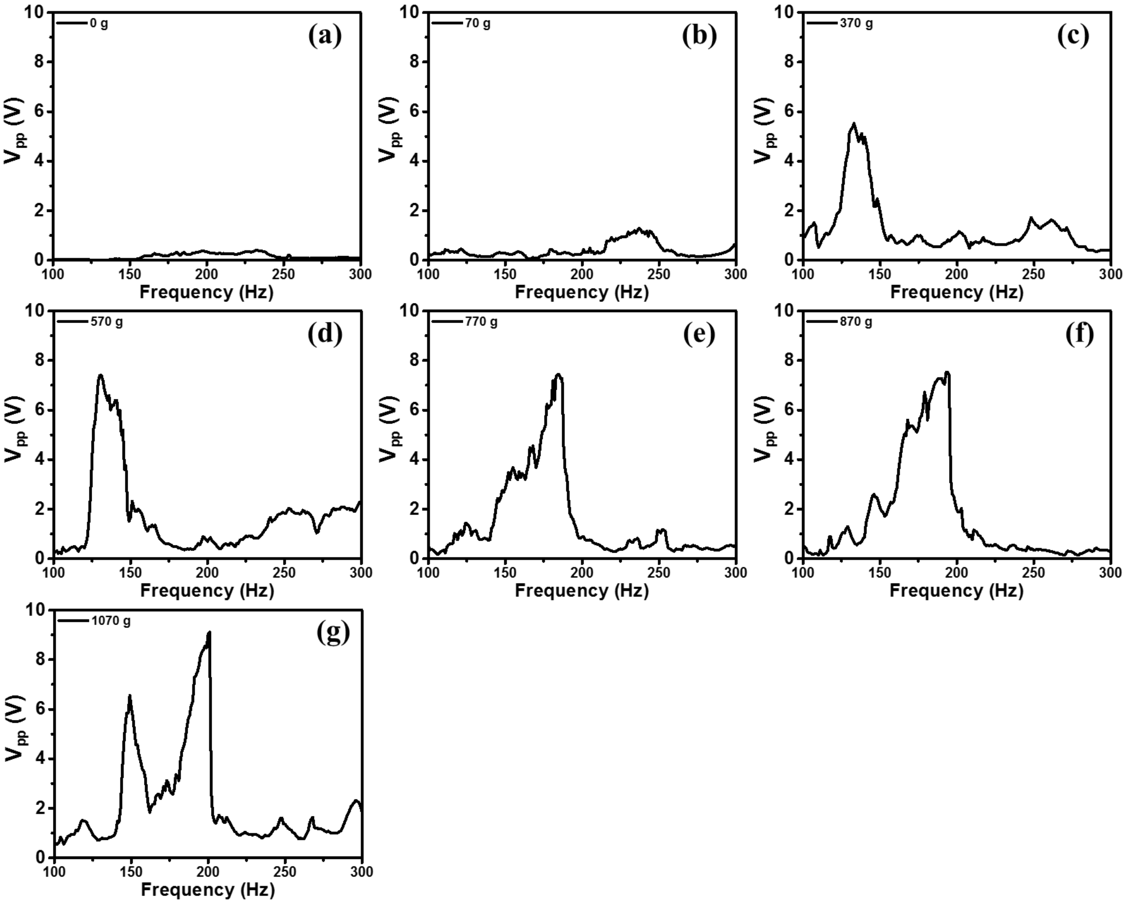

Figure S5. Increase of output voltage (V_{pp}) by stretching the M/PVDF/M generator. The applied weight is 0 g (a), 70 g (b), 370 g (c), 570 g (d), 770 g (e), 870 g (f) and 1070 g (g).

Tensile stresses were applied to the M/PVDF/M generator by hanging weights to make it stretched. The output voltages (V_{pp}) were measured as functions of the frequency of the excitation from a loudspeaker, as shown in Fig. S4. The maximum voltages (V_{pp}) at the resonance frequency were (a) 0.4 V at 232 Hz, (b) 1.3 V at 237 Hz, (c) 5.5 V at 133 Hz, (d) 7.4 V at 130 Hz, (e) 7.5V at 185 Hz, (f) 7.5 V at 194 Hz, and (g) 9.1 V at 201 Hz. Even though the resonance frequency at 0 g and 70 g seems to be aberrant, it can be considered as a spurious mode excitation due to its crumpled surface.

6. SEM, XRD and Optical microscope images of P(VDF-TrFE)

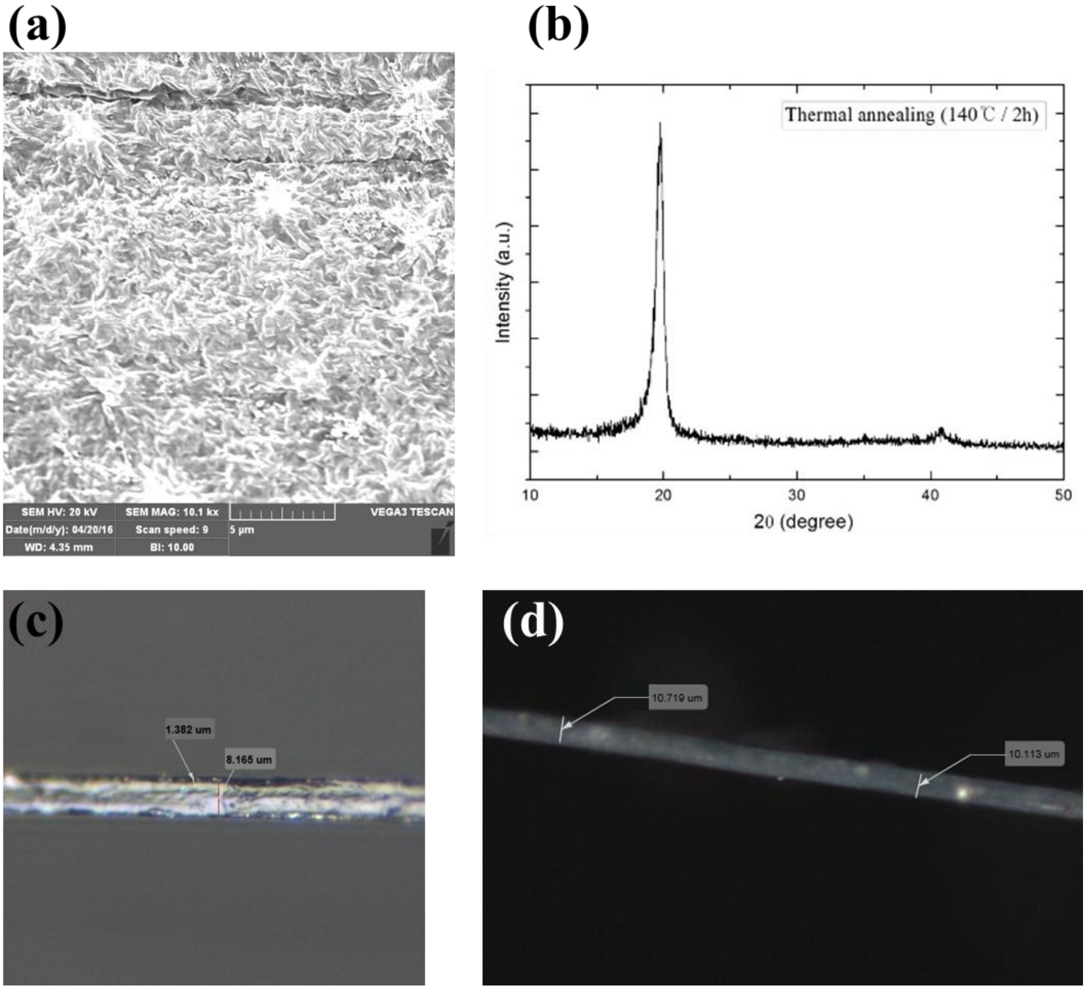

Fig. S6 (a) SEM image, (b) XRD data, and (c-d) optical microscopic images of P(VDF-TrFE) thin film are shown. XRD data indicates the β phase of the film. From optical images the thickness ~10 nm of the film was estimated.

7. Calculation of the length change by a mechanical pulling

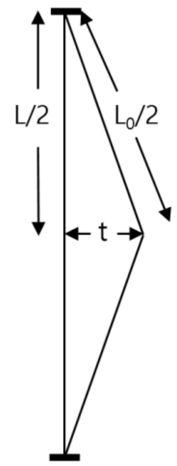

Fig. S7 Diagram for calculation of contraction clamped spring

In order to estimate the expansion of the piezo-film ΔL, the vibrating anti-node of the piezo film was measured as t = 0.7 mm as excitation of the mechanical pulses was applied. It was assumed that the shape of the expanded film was roughly similar to the triangular form as shown in Fig. S7.

$$\Delta L = L - L_0$$

$$\left(\frac{L}{2}\right)^2 = \left(\frac{L_0}{2}\right)^2 + t^2$$

$$L \cong L_0\left[1 + \frac{1}{2}\left(\frac{2t}{L_o}\right)^2\right]$$

$$\Delta L = L - L_0 \cong \frac{2t^2}{L_0}$$

From these equations, $\Delta L = 0.015$ mm was estimated.
